# Application of Isolated Mitochondria in Toxicological and Clinical Studies

**Published:** 2012

**Authors:** Jalal Pourahmad, Mir-Jamal Hosseini

Mitochondria are unique organelles with major role in cellular function, not only as a major site of ATP production, but also by regulating energy expenditure, apoptosis signaling, and production of reactive oxygen species. They play a central role in determining the point-of-no-return for the apoptotic process. There is accumulating evidence supporting a direct link between mitochondria, oxidative stress and cell death. Although we have learned a great deal about mitochondria over the last decades, the field of mitochondrial research remains a fertile one and there is no doubt that there is still much to learn about new techniques now available.

In recent years, studies from laboratories working in various aspect of mitochondria showed that Isolated mitochondria does not have the complexity of the freshly isolated cells, which have both the barrier of membrane permeability and an active metabolizing system as a difference between isolated cells and mitochondria. Now a days, analysis of mitochondrial function has become central to the basic research of mitochondrial physiology and the diagnosis of many diseases including the cancer, diabetes, cardiovascular disease, oxidative stress, and the age-related neurodegenerative diseases. Proper assessment of mitochondrial oxidative phosphorylation is essential for investigations of cellular energetics and also significantly contributes to the studies of mechanisms of apoptosis and cell death and of the pathophysiology of a variety of human diseases.

Measurements conducted by intact mitochondria isolated from fresh tissue provide distinct information regarding the function of these organelles that complements conventional mitochondrial assays using previously frozen tissues as well as in vivo assessments. On the other hand, several experiments by isolated mitochondria have revealed that the function of mitochondria remains unchanged up to 18 h following homogenization when stored at 4ºC in desired buffer. 

Mitochondrial dysfunction often occurs early in toxicological process and plays a pivotal role in determining whether cells are capable of maintaining homeostasis and repair or irreversibly damaged. Therefore, understanding of basic concepts of mitochondrial function and its role in toxicity of diverse agents, including the unique nature of the mitochondrial genome and its susceptibility to mutation, the bioactivation of compounds within the mitochondria, the detoxification systems available to necessary prevent mitochondrial dysfunction, the role of high-amplitude swelling of mitochondria in cell damage and the methodologies for measuring mitochondrial failure in association with cell injury, is highly demanded in either mechanistic or safety assessment studies associated with new drug development.

It is noteworthy that there are different types of mitochondrial toxicants and therefore different types of mechanisms involved in mitochondrial dysfunction via interference with their oxidative phosphorylation, bio-activation of compounds or generating electrophile intermediates and finally damaging the mitochondrial DNA. It is therefore suggested that using of isolated mitochondria is suitable for assessment of mitochondrial dysfunction and it also perfectly provides information regarding failure of energy metabolism, calcium homeostasis and finally detoxication and repair processes in corresponding intact cells. Advances in mitochondrial research in medical technology have been a major impetus behind the desire to design and develop drugs specifically targeting mitochondria for therapeutic gain. The potential therapeutic applications of mitochondrial targeting include: (a) delivery of antioxidants to mitochondria to prevent oxidative damage associated with the neurodegenerative diseases, ischaemia and reperfusion injuries and diabetes; (b) targeting of toxic drugs to mitochondria to trigger apoptosis in cancer therapy; (c) delivery of drugs to mitochondria to inhibit the mitochondrial permeability transition (MPT) in ischaemia and reperfusion -related tissue injuries, for example, in heart attack and stroke; and (d) the targeting of drugs to either uncouple the electron transport chain (ETC), or activate the uncoupling proteins (UCPs), in obesity and diabetes.

*Jalal Pourahmad is currently a Professor at Department of Pharmacology and Toxicology, School of Pharmacy, Shahid Beheshti University of Medical Sciences Tehran, Iran. He may be reached at e-mail address:* j.pourahmadjaktaji@utoronto.ca

*Mir-Jamal Hosseini is currently an Assistant Professor at Department of Pharmacology and Toxicology, School of Pharmacy zanjan University of Medical Sciences zanjan, Iran. He may be reached at e-mail address:jamal_hossini@yahoo.com*

